# Bis(μ_2_-*N*-methyl-*N*-phenyl­dithio­carbamato)-κ^3^ *S*,*S*′:*S*;κ^3^ *S*:*S*,*S*′-bis­[(*N*-methyl-*N*-phenyl­dithio­carbamato-κ^2^ *S*,*S*′)cadmium]: crystal structure and Hirshfeld surface analysis

**DOI:** 10.1107/S2056989017002705

**Published:** 2017-02-21

**Authors:** Siti Aisyah Nabilah Suwardi, See Mun Lee, Kong Mun Lo, Mukesh M. Jotani, Edward R. T. Tiekink

**Affiliations:** a Department of Chemistry, University of Malaya, 50603 Kuala Lumpur, Malaysia; b Research Centre for Crystalline Materials, School of Science and Technology, Sunway University, 47500 Bandar Sunway, Selangor Darul Ehsan, Malaysia; c Department of Physics, Bhavan’s Sheth R. A. College of Science, Ahmedabad, Gujarat 380 001, India

**Keywords:** crystal structure, cadmium, di­thio­carbamate, Hirshfeld surface analysis

## Abstract

The title compound, [Cd_2_(C_8_H_8_NS_2_)_4_], is a centrosymmetric dimer with both chelating and μ_2_-tridentate di­thio­carbamate ligands. The resulting S_5_ donor set defines a Cd^II^ coordination geometry inter­mediate between square-pyramidal and trigonal–bipyramidal, but tending towards the former. The packing features C—H⋯S and C—H⋯π inter­actions, which generate a three-dimensional network. The influence of these inter­actions, along with intra-dimer π–π inter­actions between chelate rings, has been investigated by an analysis of the Hirshfeld surface.

## Abstract 2

With both chelating and μ_2_-tridentate di­thio­carbamate ligands, the structure of [Cd_2_(C_8_H_8_NS_2_)_4_] is a centrosymmetric dimer. The packing features C—H⋯S and C—H⋯π inter­actions.

## Chemical context   

The structural chemistry of the binary zinc-triad (group 12) di­thio­carbamates (^−^S_2_CN*RR*′)_2_ (*R*/*R*′ = alk­yl/aryl), along with related 1,1-di­thiol­ate ligands, *i.e.* di­thio­phosphates [^−^S_2_P(O*R*)_2_] and di­thio­carbonates (xanthates; ^−^S_2_CO*R*), have long attracted the attention of structural chemists owing to their diversity of structures/supra­molecular association patterns in the solid state (Cox & Tiekink, 1997 ▸; Tiekink, 2003 ▸). The common structural motif adopted by all elements is one that features two chelating ligands and two tridentate ligands (chelating one metal atom and simultaneously bridging to a second), leading, usually, to a centrosymmetric binuclear mol­ecule. Indeed, most zinc di­thio­carbamate structures adopt this motif, but when the *R*/*R*′ are bulky, a mononuclear species with tetrahedrally coordinated zinc atoms is found; significantly greater structural variety has been noted for the binary zinc di­thio­phosphates and xanthates (Lai *et al.*, 2002 ▸; Tan *et al.*, 2015 ▸). More diversity in structural motifs is noted in the binary cadmium di­thio­carbamates with the recent observation of linear polymeric forms with hexa­coordinated cadmium atoms (Tan *et al.*, 2013 ▸, 2016 ▸; Ferreira *et al.*, 2016 ▸). Systematic studies indicated solvent-mediated transformations between polymeric and binuclear structural motifs, with the latter being the thermodynamically more stable (Tan *et al.*, 2013 ▸, 2016 ▸). The greatest structural diversity among the zinc-triad di­thio­carbamates is found for the binary mercury compounds, where mononuclear, binuclear and polymeric structures have been observed, as summarized very recently (Jotani *et al.*, 2016 ▸). Complementing the structural motifs already mentioned for zinc and cadmium is a trinuclear species, {Hg[S_2_CN(tetra­hydro­quinoline)]_2_}_3_ (Rajput *et al.*, 2014 ▸), with the central Hg^II^ atom being hexa­coordinated, as in the polymeric form, and the peripheral Hg^II^ atoms being coordinated as in the binuclear form, indicating the possibility that this is an inter­mediate metastable form in the crystallization of this compound. In light of the above, when crystals of the title compound became available, namely {Cd[S_2_CN(Me)Ph]_2_}_2_, (I), its crystal and mol­ecular structures were studied, along with an evaluation of the supra­molecular association in the crystal through an analysis of the Hirshfeld surface.
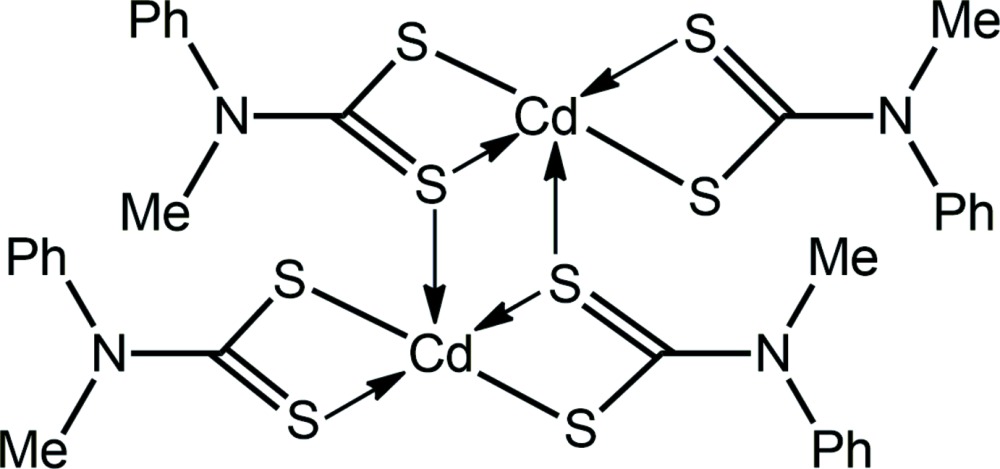


## Structural commentary   

**Figure 1:**
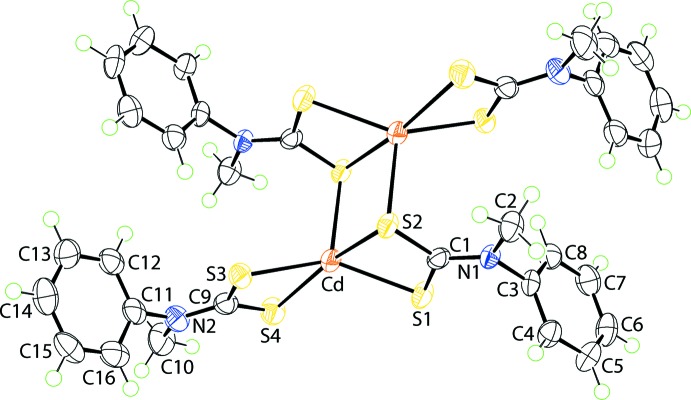
The mol­ecular structure of (I), showing the atom-labelling scheme and displacement ellipsoids at the 70% probability level. The mol­ecule is located about a centre of inversion and unlabelled atoms are generated by the symmetry operation (−*x*, 1 − *y*, 1 − *z*).

The centrosymmetric binuclear mol­ecule of (I) (Fig. 1 ▸) conforms to the common binuclear motif adopted by binary zinc-triad di­thio­carbamates. The S1 di­thio­carbamate anion forms a nearly symmetric bridge, as seen in the value of Δ(Cd—S) = 0.09 Å = Cd—S_long_ − Cd—S_short_. Within the resultant {CdSCS}_2_ eight-membered ring, which adopts a chair conformation, the bridging S2 atom also forms a longer [S2—Cd^i^ = 2.9331 (8) Å; symmetry code: (i) −*x*, 1 − *y*, 1 − *z*] transannular inter­action. The S3 di­thio­carbamate ligand is strictly chelating, with Δ(Cd—S) = 0.08 Å. Reflecting the symmetric modes of coordination of the di­thio­carbamate ligands, the C—S bond lengths are equal within 5σ (Table 1 ▸).

The resultant S_5_ donor set defines a highly distorted penta­coordinate geometry, with the major distortions due to the disparate Cd—S bond lengths and the acute angles subtended at the Cd^II^ atom by the chelating ligands (Table 1 ▸). The widest angle at the Cd^II^ atom involves the S atoms forming the weaker Cd—S inter­actions, *i.e*. S2—Cd—S4 = 161.85 (3)°. A measure of the distortion of a coordination geometry from the ideal square-pyramidal and trigonal–bipyramidal geometries is given by the value of τ (Addison *et al.*, 1984 ▸), which computes to 0.0 and 1.0 for the ideal geometries, respectively. In (I), the value of τ is 0.39, *i.e*. inter­mediate between the two extremes, but tending towards the former.

## Supra­molecular features   

**Figure 2:**
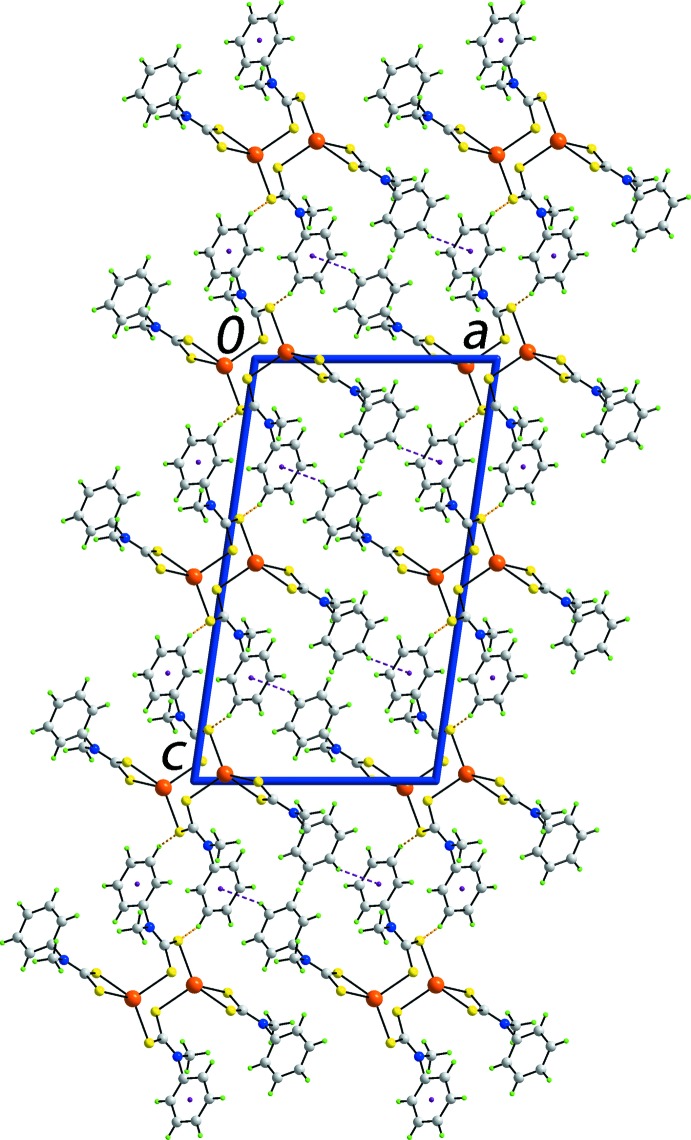
A view of the unit-cell contents of (I) in projection down the *b* axis. The C—H⋯π(chelate ring) and C—H⋯S inter­actions are shown as purple and orange dashed lines, respectively.

Two specific inter­molecular inter­actions have been identified in the mol­ecular packing of (I), and each involves the participation of phenyl ring C3–C8 (Table 2 ▸). Phenyl-C—H⋯π inter­actions with the C3–C8 ring as the acceptor lead to supra­molecular layers parallel to (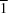
02), as each binuclear mol­ecule participates in four such inter­actions. The layers are connected into a three-dimensional architecture by phenyl-C—H⋯S inter­actions, *i.e*. with the C3–C8 ring as donor (Fig. 2 ▸).

## Hirshfeld surface analysis   

**Figure 3:**
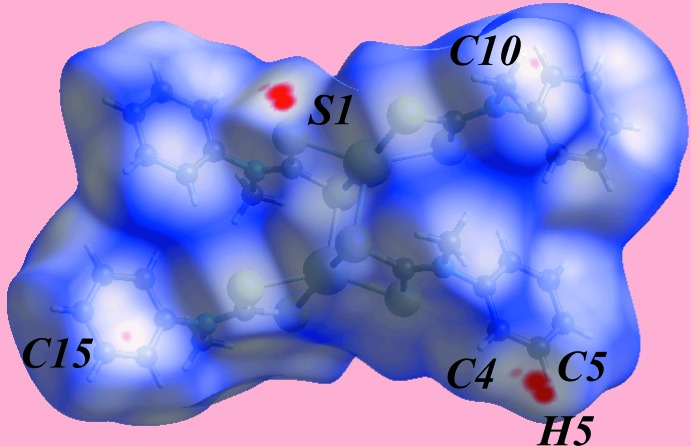
A view of the Hirshfeld surface for (I) mapped over *d*_norm_ in the range −0.055 to 1.371 au.

**Figure 4:**
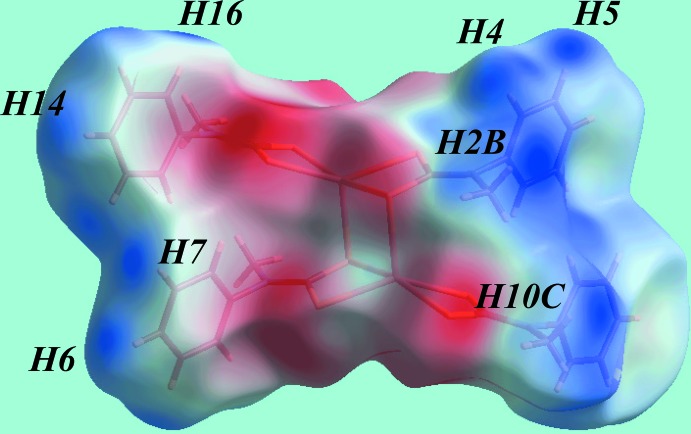
A view of Hirshfeld surface for (I) mapped over the electrostatic potential in the range ±0.048 au.

**Figure 5:**
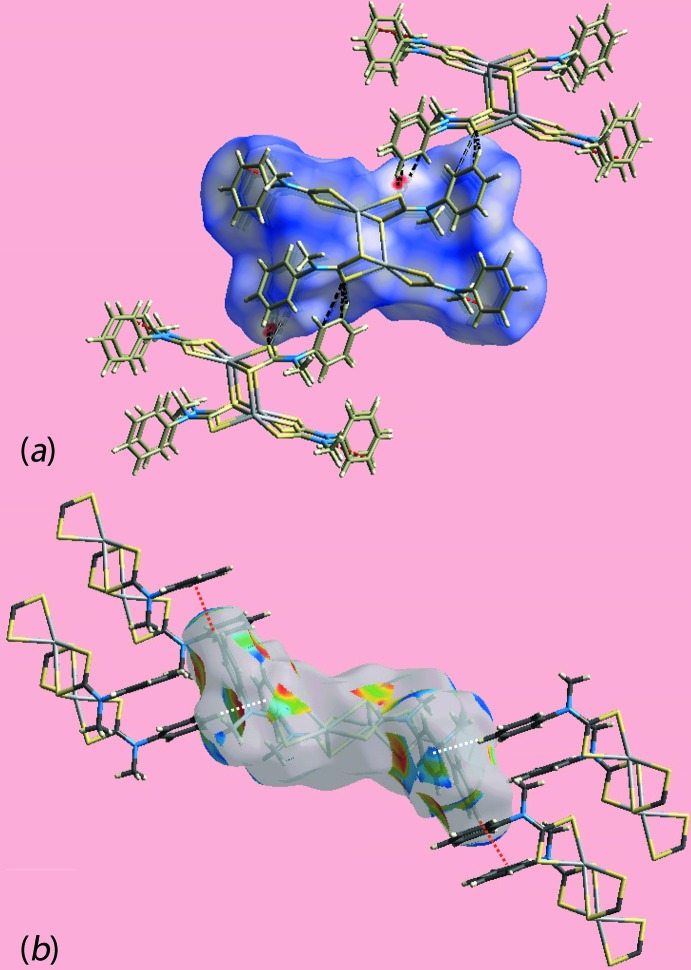
Views of the Hirshfeld surface mapped over (*a*) *d*_norm_ about a reference mol­ecule, highlighting the inter­molecular C—H⋯S inter­actions and short inter­atomic C⋯C contacts as black and red dashed lines, respectively, and (*b*) with shape-index property about a reference mol­ecule. The C—H⋯π and π⋯H—C inter­actions are indicated with red and white dotted lines, respectively.

The Hirshfeld surface analysis for (I) was performed as described in a recent report of a related binuclear cadmium di­thio­carbamate compound (Jotani *et al.*, 2016 ▸). On the Hirshfeld surface mapped over *d*_norm_ in the range −0.055 to 1.371 au (Fig. 3 ▸), the bright-red spots near the C5, H5 and S1 atoms indicate respective donors and acceptors of inter­molecular C—H⋯S inter­actions; the other pair of faint-red spots near atoms C4 and S1 represent a weaker inter­action (Table 3 ▸). The donors and acceptors of the specified C—H⋯S and C—H⋯π inter­actions in Table 2 ▸, and short inter­atomic C⋯H/H⋯C contacts (Table 3 ▸) give rise to positive and negative potentials, respectively, and are viewed as the blue and red regions on Hirshfeld surface mapped over electrostatic potential (in the range ±0.048 au) (Fig. 4 ▸). The immediate environments about a reference mol­ecule within *d*_norm_ and shape-index mapped Hirshfeld surface are illustrated in Figs. 5 ▸(*a*) and 5(*b*), respectively, and again highlight the influence of C—H⋯S inter­actions, short C10⋯C15 contacts and C—H⋯π inter­actions involving phenyl rings (atoms C3–C8) as the acceptor. Thus, the C—H⋯S inter­actions involving the phenyl-ring C4, C5 and H5 atoms with S1 are shown with black dashed lines in Fig. 5 ▸(*a*); the red dashed lines indicate short inter­atomic C⋯C contacts (Table 3 ▸). The C—H⋯π and their reciprocal contacts, *i.e*. π⋯H—C, with phenyl-ring atom C14 as donor and phenyl ring C3–C8 as acceptor, are shown with red and white dotted lines, respectively, on the Hirshfeld surface mapped with shape-index property in Fig. 5 ▸(*b*).

**Figure 6:**
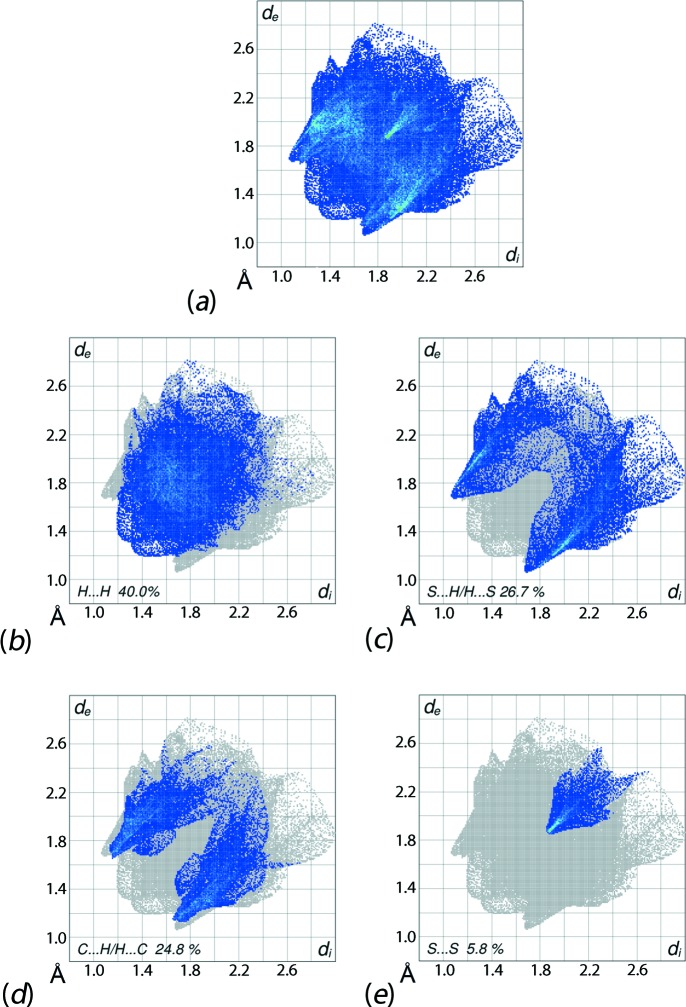
Fingerprint plots for (I): (*a*) overall and those delineated into (*b*) H⋯H, (*c*) S⋯H/H⋯S, (*d*) C⋯H/H⋯C and (*e*) S⋯S contacts.

The overall two-dimensional fingerprint plot and those delineated into H⋯H, S⋯H/H⋯S, C⋯H/H⋯C and S⋯S contacts (McKinnon *et al.*, 2007 ▸) are illustrated in Figs. 6 ▸(*a*)–(*e*); their relative contributions to the Hirshfeld surface are summarized qu­anti­tatively in Table 4 ▸. The relatively low contribution of H⋯H contacts to the Hirshfeld surface results from the involvement of surface H atoms in inter­molecular C—H⋯S, C—H⋯π and C⋯H/H⋯C contacts. It is apparent from the fingerprint plot delineated into H⋯H contacts (Fig. 6 ▸ *b*) that H⋯H contacts do not exert much influence on the mol­ecular packing, as their inter­atomic distances are greater than the sum of their van der Waals radii, *i.e. d*_e_ + *d*_i_ > 2.8 Å. A pair of peaks appearing in the fingerprint plot delineated into S⋯H/H⋯S contacts at *d*_e_ + *d*_i_ ∼ 2.8 Å (Fig. 6 ▸ *c*) arise from the C5—H5⋯S1 inter­action; the weaker C4⋯H4⋯S1 inter­action and short inter­atomic H⋯S/S⋯H contacts involving the S3 atom (Table 3 ▸) are viewed as a pair of thin green lines aligned at *d*_e_ + *d*_i_ ∼ 2.9 Å.

The distribution of points showing the superimposition of a forceps-like shape on characteristic wings in the fingerprint plot delineated into C⋯H/H⋯C contacts (Fig. 6 ▸ *d*) indicate the significance of these contacts through the presence of C—H⋯π inter­actions and short inter­atomic C⋯H/H⋯C contacts in the crystal. A pair of green lines within the forceps also indicates the influence of these contacts. Finally, an arrow-shaped distribution of green points in the centre in the plot corresponding to S⋯S contacts (Fig. 6 ▸ *e*), together with the contribution from Cd⋯S/S⋯Cd contacts to the Hirshfeld surface (Table 4 ▸), show the presence of intra­molecular π–π stacking inter­actions between the Cd/S1/C1/S2 chelate rings of inversion-related mol­ecules [*Cg*⋯*Cg* = 3.6117 (11) Å; symmetry code: −*x*, 1 − *y*, 1 − *z*]. The small contributions from Cd⋯H/H⋯Cd and N⋯H/H⋯N contacts (Table 4 ▸) do not impact significantly on the mol­ecular packing.

## Database survey   

The di­thio­carbamate ligand featured in (I) has been reported in several other crystal structures (Groom *et al.*, 2016 ▸). Indeed, the binary zinc (Baba *et al.*, 2002 ▸) and mercury (Onwudiwe & Ajibade, 2011*a* ▸,*b* ▸) structures have been reported already, so, in this sense, the structure of (I) completes the series. The zinc compound adopts the common binuclear motif (Baba *et al.*, 2002 ▸). More inter­esting is the fact that for the mercury structure, both mononuclear (Onwudiwe & Ajibade, 2011*a* ▸) and binuclear (Onwudiwe & Ajibade, 2011*b* ▸) forms have been reported (Tan *et al.*, 2015 ▸). As to the other main group element structures, the binary di­thio­carbamate compounds of anti­mony(III) (Baba *et al.*, 2003 ▸) and bis­muth(III) (Yin *et al.*, 2004 ▸), including an aceto­nitrile solvate (Lai & Tiekink, 2007 ▸), have been described. These, too, present the same structural features as reported for the overwhelming majority of related anti­mony(III) (Liu & Tiekink, 2005 ▸) and bis­muth(III) di­thio­carbamate compounds (Lai & Tiekink, 2007 ▸).

## Synthesis and crystallization   

All chemicals and solvents were used as purchased without purification, and all reactions were carried out under ambient conditions. The melting point was determined using an Electrothermal digital melting-point apparatus and was uncorrected. The IR spectrum was obtained on a PerkinElmer Spectrum 400 FT Mid-IR/Far-IR spectrophotometer from 4000 to 400 cm^−1^. ^1^H and ^13^C NMR spectra were recorded at room temperature in DMSO-*d*_6_ solution on a Jeol ECA 400 MHz FT–NMR spectrometer.

Sodium methyl­phenyl­dithio­carbamate (1.0 mmol, 0.205 g) in methanol (25 ml) was added to cadmium chloride (1.0 mmol, 0.183 g) in methanol (10 ml). The resulting mixture was stirred and refluxed for 2 h. The filtrate was evaporated until an off-white precipitate was obtained, which was recrystallized in methanol. Slow evaporation of the filtrate yielded colourless crystals of the title compound (yield: 0.194 g, 61%; m.p. 473 K). IR (cm^−1^): 1491 (*m*) [ν(C—N)], 1160 (*m*), 964 (*s*) [ν(C—S)] cm^−1^. ^1^H NMR: δ 7.26–7.42 (*m*, 5H, aromatic H), 2.05 (*s*, 3H, CH_3_). ^13^C NMR: δ 46.6 (Me) 125.6, 128.4, 129.6, 147.9 (aromatic C), 207.8 (CS_2_).

## Refinement   

Crystal data, data collection and structure refinement details are summarized in Table 5 ▸. Carbon-bound H atoms were placed in calculated positions (C—H = 0.95–0.98 Å) and were included in the refinement in the riding-model approximation, with *U*_iso_(H) values set at 1.2–1.5*U*_eq_(C).

## Supplementary Material

Crystal structure: contains datablock(s) I, global. DOI: 10.1107/S2056989017002705/hb7659sup1.cif

Structure factors: contains datablock(s) I. DOI: 10.1107/S2056989017002705/hb7659Isup2.hkl

CCDC reference: 1533246

Additional supporting information:  crystallographic information; 3D view; checkCIF report

## Figures and Tables

**Table 1:** Selected geometric parameters (Å, °)

Cd—S1	2.5044 (8)	C1—S2	1.739 (3)
Cd—S2	2.9331 (8)	C9—S3	1.730 (3)
Cd—S2^i^	2.5942 (8)	C9—S4	1.717 (4)
Cd—S3	2.5397 (9)	C1—N1	1.326 (4)
Cd—S4	2.6196 (8)	C9—N2	1.344 (4)
C1—S1	1.716 (3)		
			
S1—Cd—S2	66.15 (2)	S2—Cd—S4	161.85 (3)
S1—Cd—S3	138.16 (3)	S2—Cd—S2^i^	92.58 (2)
S1—Cd—S4	114.48 (3)	S3—Cd—S4	70.93 (3)
S1—Cd—S2^i^	104.42 (3)	S3—Cd—S2^i^	114.47 (3)
S2—Cd—S3	96.36 (2)	S4—Cd—S2^i^	104.38 (3)

Symmetry code: (i) 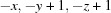.

**Table 2:** Hydrogen-bond geometry (Å, °) *Cg*1 is the centroid of the C3–C8 ring.

*D*—H⋯*A*	*D*—H	H⋯*A*	*D*⋯*A*	*D*—H⋯*A*
C14—H14⋯*Cg*1^ii^	0.95	2.99	3.883 (4)	156
C5—H5⋯S1^iii^	0.95	2.75	3.372 (4)	124

Symmetry codes: (ii) 
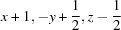
; (iii) 
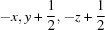
.

**Table 3:** Short inter­atomic contacts (Å) in (I)

Contact	Distance	Symmetry operation
S1⋯C4	3.462 (3)	−*x*, − 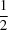 + *y*, −*z*
S1⋯H4	2.94	−*x*, − 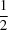 + *y*, −*z*
S3⋯H16	2.88	1 − *x*, 1 − *y*, 1 − *z*
C10⋯C15	3.376 (5)	*x*, −1 + *y*, *z*
C7⋯H2*B*	2.89	*x*, −1 + *y*, *z*
C13⋯H7	2.84	1 − *x*, −*y*, *z*
C14⋯H7	2.87	1 − *x*, −*y*, *z*
C14⋯H10*C*	2.81	*x*, 1 + *y*, *z*
C15⋯H6	2.84	1 − *x*, −*y*, *z*

**Table 4:** Percentage contributions of the different inter­molecular contacts to the Hirshfeld surface in (I)

Contact	% Contribution in (I)
H⋯H	40.0
S⋯H/H⋯S	26.7
C⋯H/H⋯C	24.8
S⋯S	5.8
Cd⋯H/H⋯Cd	1.2
N⋯H/H⋯N	0.8
Cd⋯S/S⋯Cd	0.7

**Table 5:** Experimental details

Crystal data	
Chemical formula	[Cd_2_(C_8_H_8_NS_2_)_4_]
*M*_r_	953.92
Crystal system, space group	Monoclinic, *P*2_1_/*c*
Temperature (K)	100
*a*, *b*, *c* (Å)	12.7972 (6), 6.4445 (3), 22.582 (1)
β (°)	98.247 (4)
*V* (Å^3^)	1843.11 (15)
*Z*	2
Radiation type	Mo *K*α
μ (mm^−1^)	1.64
Crystal size (mm)	0.20 × 0.15 × 0.10

Data collection	
Diffractometer	Agilent SuperNova Dual Source diffractometer with an Atlas detector
Absorption correction	Multi-scan (*CrysAlis PRO*; Agilent, 2013 ▸)
*T*_min_, *T*_max_	0.731, 1.000
No. of measured, independent and observed [*I* > 2σ(*I*)] reflections	11881, 4894, 3804
*R*_int_	0.037
(sin θ/λ)_max_ (Å^−1^)	0.708

Refinement	
*R*[*F* ^2^ > 2σ(*F* ^2^)], *wR*(*F* ^2^), *S*	0.037, 0.086, 1.05
No. of reflections	4894
No. of parameters	210
H-atom treatment	H-atom parameters constrained
Δρ_max_, Δρ_min_ (e Å^−3^)	0.72, −0.48

Computer programs: *CrysAlis PRO* (Agilent, 2013 ▸), *SHELXS97* (Sheldrick, 2008 ▸), *SHELXL2014* (Sheldrick, 2015 ▸), *ORTEP-3 for Windows* (Farrugia, 2012 ▸), *DIAMOND* (Brandenburg, 2006 ▸) and *publCIF* (Westrip, 2010 ▸).

## supplementary crystallographic information

### Crystal data

**Table:**

[Cd_2_(C_8_H_8_NS_2_)_4_]	*F*(000) = 952
*M**_r_* = 953.92	*D*_x_ = 1.719 Mg m^−^^3^
Monoclinic, *P*2_1_/*c*	Mo *K*α radiation, λ = 0.71073 Å
*a* = 12.7972 (6) Å	Cell parameters from 4387 reflections
*b* = 6.4445 (3) Å	θ = 3.4–29.8°
*c* = 22.582 (1) Å	µ = 1.64 mm^−^^1^
β = 98.247 (4)°	*T* = 100 K
*V* = 1843.11 (15) Å^3^	Block, colourless
*Z* = 2	0.20 × 0.15 × 0.10 mm

### Data collection

**Table:**

Agilent SuperNova Dual Source diffractometer with an Atlas detector	4894 independent reflections
Radiation source: SuperNova (Mo) X-ray Source	3804 reflections with *I* > 2σ(*I*)
Mirror monochromator	*R*_int_ = 0.037
Detector resolution: 10.4041 pixels mm^-1^	θ_max_ = 30.2°, θ_min_ = 3.2°
ω scan	*h* = −12→17
Absorption correction: multi-scan (CrysAlis PRO; Agilent, 2013)	*k* = −9→8
*T*_min_ = 0.731, *T*_max_ = 1.000	*l* = −29→30
11881 measured reflections	

### Refinement

**Table:**

Refinement on *F*^2^	Primary atom site location: structure-invariant direct methods
Least-squares matrix: full	Hydrogen site location: inferred from neighbouring sites
*R*[*F*^2^ > 2σ(*F*^2^)] = 0.037	H-atom parameters constrained
*wR*(*F*^2^) = 0.086	*w* = 1/[σ^2^(*F*_o_^2^) + (0.0331*P*)^2^ + 0.5876*P*] where *P* = (*F*_o_^2^ + 2*F*_c_^2^)/3
*S* = 1.05	(Δ/σ)_max_ = 0.001
4894 reflections	Δρ_max_ = 0.72 e Å^−^^3^
210 parameters	Δρ_min_ = −0.48 e Å^−^^3^
0 restraints	

### Special details

**Table:**

Geometry. All esds (except the esd in the dihedral angle between two l.s. planes) are estimated using the full covariance matrix. The cell esds are taken into account individually in the estimation of esds in distances, angles and torsion angles; correlations between esds in cell parameters are only used when they are defined by crystal symmetry. An approximate (isotropic) treatment of cell esds is used for estimating esds involving l.s. planes.

### Fractional atomic coordinates and isotropic or equivalent isotropic displacement parameters (Å^2^)

**Table:**

	*x*	*y*	*z*	*U*_iso_*/*U*_eq_	
Cd	0.12343 (2)	0.35538 (4)	0.48383 (2)	0.02641 (8)	
S1	0.02923 (7)	0.37408 (12)	0.37881 (4)	0.02834 (19)	
S2	0.02121 (6)	0.75614 (12)	0.45307 (3)	0.02341 (17)	
S3	0.28949 (6)	0.50187 (12)	0.54452 (4)	0.02566 (18)	
S4	0.26597 (7)	0.06179 (13)	0.50220 (4)	0.0311 (2)	
N1	−0.09657 (19)	0.6884 (4)	0.34672 (11)	0.0205 (5)	
N2	0.4316 (2)	0.2090 (4)	0.57566 (12)	0.0287 (6)	
C1	−0.0239 (2)	0.6135 (5)	0.38915 (13)	0.0220 (6)	
C2	−0.1463 (3)	0.8929 (5)	0.34931 (15)	0.0286 (7)	
H2A	−0.2198	0.8754	0.3559	0.043*	
H2B	−0.1443	0.9663	0.3115	0.043*	
H2C	−0.1078	0.9736	0.3823	0.043*	
C3	−0.1353 (2)	0.5650 (5)	0.29431 (13)	0.0217 (6)	
C4	−0.0939 (3)	0.5954 (5)	0.24187 (14)	0.0271 (7)	
H4	−0.0392	0.6940	0.2401	0.033*	
C5	−0.1334 (3)	0.4800 (5)	0.19168 (15)	0.0314 (8)	
H5	−0.1055	0.4999	0.1553	0.038*	
C6	−0.2119 (3)	0.3383 (5)	0.19425 (16)	0.0334 (8)	
H6	−0.2381	0.2593	0.1598	0.040*	
C7	−0.2535 (3)	0.3093 (5)	0.24708 (17)	0.0334 (8)	
H7	−0.3084	0.2111	0.2487	0.040*	
C8	−0.2150 (2)	0.4235 (5)	0.29753 (15)	0.0271 (7)	
H8	−0.2432	0.4045	0.3338	0.032*	
C9	0.3372 (2)	0.2509 (5)	0.54363 (14)	0.0257 (7)	
C10	0.4776 (3)	0.0001 (5)	0.57988 (17)	0.0398 (9)	
H10A	0.5511	0.0070	0.5724	0.060*	
H10B	0.4372	−0.0902	0.5500	0.060*	
H10C	0.4752	−0.0560	0.6200	0.060*	
C11	0.4909 (2)	0.3640 (5)	0.61192 (15)	0.0278 (7)	
C12	0.4636 (3)	0.4133 (5)	0.66742 (15)	0.0303 (7)	
H12	0.4045	0.3484	0.6808	0.036*	
C13	0.5227 (3)	0.5572 (6)	0.70328 (16)	0.0345 (8)	
H13	0.5038	0.5920	0.7412	0.041*	
C14	0.6088 (3)	0.6503 (5)	0.68420 (17)	0.0378 (9)	
H14	0.6491	0.7494	0.7089	0.045*	
C15	0.6364 (3)	0.5989 (6)	0.62904 (18)	0.0367 (9)	
H15	0.6965	0.6615	0.6162	0.044*	
C16	0.5770 (3)	0.4566 (5)	0.59235 (16)	0.0329 (8)	
H16	0.5953	0.4232	0.5542	0.039*	

### Atomic displacement parameters (Å^2^)

**Table:**

	*U*^11^	*U*^22^	*U*^33^	*U*^12^	*U*^13^	*U*^23^
Cd	0.02523 (14)	0.03538 (16)	0.01777 (12)	0.00772 (10)	0.00019 (9)	0.00105 (10)
S1	0.0369 (5)	0.0274 (4)	0.0186 (4)	0.0104 (4)	−0.0034 (3)	−0.0032 (3)
S2	0.0267 (4)	0.0251 (4)	0.0172 (4)	0.0002 (3)	−0.0014 (3)	−0.0024 (3)
S3	0.0252 (4)	0.0258 (4)	0.0251 (4)	0.0074 (3)	0.0005 (3)	0.0023 (3)
S4	0.0325 (5)	0.0277 (4)	0.0319 (5)	0.0082 (4)	0.0001 (3)	−0.0026 (4)
N1	0.0237 (13)	0.0209 (13)	0.0161 (12)	0.0013 (10)	−0.0002 (10)	0.0015 (10)
N2	0.0266 (14)	0.0273 (14)	0.0315 (16)	0.0099 (12)	0.0017 (12)	0.0024 (12)
C1	0.0239 (16)	0.0259 (17)	0.0168 (15)	−0.0029 (13)	0.0051 (12)	0.0014 (12)
C2	0.0355 (19)	0.0213 (16)	0.0271 (18)	0.0041 (14)	−0.0019 (14)	0.0021 (13)
C3	0.0238 (15)	0.0208 (15)	0.0184 (15)	0.0022 (13)	−0.0039 (11)	0.0015 (12)
C4	0.0293 (17)	0.0289 (17)	0.0221 (16)	−0.0056 (14)	0.0000 (13)	0.0004 (13)
C5	0.042 (2)	0.0311 (18)	0.0196 (16)	−0.0015 (16)	−0.0005 (14)	0.0018 (14)
C6	0.0358 (19)	0.0315 (19)	0.0285 (19)	−0.0031 (15)	−0.0106 (14)	−0.0058 (15)
C7	0.0269 (18)	0.0319 (18)	0.039 (2)	−0.0059 (15)	−0.0027 (14)	−0.0007 (16)
C8	0.0249 (16)	0.0290 (17)	0.0267 (17)	0.0022 (14)	0.0017 (13)	0.0026 (14)
C9	0.0256 (17)	0.0294 (18)	0.0230 (16)	0.0072 (14)	0.0059 (12)	0.0042 (14)
C10	0.041 (2)	0.033 (2)	0.042 (2)	0.0175 (16)	−0.0041 (16)	0.0032 (17)
C11	0.0216 (16)	0.0307 (18)	0.0301 (18)	0.0101 (14)	0.0002 (13)	0.0079 (14)
C12	0.0242 (17)	0.0356 (19)	0.0309 (19)	0.0053 (15)	0.0030 (14)	0.0096 (15)
C13	0.0335 (19)	0.039 (2)	0.0290 (19)	0.0069 (16)	−0.0007 (15)	0.0040 (16)
C14	0.033 (2)	0.033 (2)	0.043 (2)	0.0031 (16)	−0.0094 (16)	0.0071 (17)
C15	0.0211 (17)	0.038 (2)	0.051 (2)	0.0048 (15)	0.0048 (15)	0.0161 (17)
C16	0.0290 (18)	0.0348 (19)	0.036 (2)	0.0103 (16)	0.0079 (15)	0.0112 (16)

### Geometric parameters (Å, º)

**Table:**

Cd—S1	2.5044 (8)	C4—H4	0.9500
Cd—S2	2.9331 (8)	C5—C6	1.365 (5)
Cd—S2^i^	2.5942 (8)	C5—H5	0.9500
Cd—S3	2.5397 (9)	C6—C7	1.387 (5)
Cd—S4	2.6196 (8)	C6—H6	0.9500
C1—S1	1.716 (3)	C7—C8	1.386 (5)
C1—S2	1.739 (3)	C7—H7	0.9500
S2—Cd^i^	2.5942 (8)	C8—H8	0.9500
C9—S3	1.730 (3)	C10—H10A	0.9800
C9—S4	1.717 (4)	C10—H10B	0.9800
C1—N1	1.326 (4)	C10—H10C	0.9800
N1—C3	1.453 (4)	C11—C16	1.380 (5)
N1—C2	1.468 (4)	C11—C12	1.386 (5)
C9—N2	1.344 (4)	C12—C13	1.383 (5)
N2—C11	1.438 (4)	C12—H12	0.9500
N2—C10	1.467 (4)	C13—C14	1.377 (5)
C2—H2A	0.9800	C13—H13	0.9500
C2—H2B	0.9800	C14—C15	1.383 (5)
C2—H2C	0.9800	C14—H14	0.9500
C3—C8	1.378 (4)	C15—C16	1.387 (5)
C3—C4	1.379 (4)	C15—H15	0.9500
C4—C5	1.389 (4)	C16—H16	0.9500
			
S1—Cd—S2	66.15 (2)	C6—C5—H5	119.8
S1—Cd—S3	138.16 (3)	C4—C5—H5	119.8
S1—Cd—S4	114.48 (3)	C5—C6—C7	120.1 (3)
S1—Cd—S2^i^	104.42 (3)	C5—C6—H6	119.9
S2—Cd—S3	96.36 (2)	C7—C6—H6	119.9
S2—Cd—S4	161.85 (3)	C8—C7—C6	120.0 (3)
S2—Cd—S2^i^	92.58 (2)	C8—C7—H7	120.0
S3—Cd—S4	70.93 (3)	C6—C7—H7	120.0
S3—Cd—S2^i^	114.47 (3)	C3—C8—C7	119.1 (3)
S4—Cd—S2^i^	104.38 (3)	C3—C8—H8	120.5
C1—S1—Cd	93.49 (11)	C7—C8—H8	120.5
C1—S2—Cd^i^	97.54 (10)	N2—C9—S4	121.1 (2)
C1—S2—Cd	79.34 (11)	N2—C9—S3	118.3 (3)
Cd^i^—S2—Cd	87.43 (2)	S4—C9—S3	120.62 (19)
C9—S3—Cd	85.16 (11)	N2—C10—H10A	109.5
C9—S4—Cd	82.93 (11)	N2—C10—H10B	109.5
C1—N1—C3	120.7 (3)	H10A—C10—H10B	109.5
C1—N1—C2	124.1 (3)	N2—C10—H10C	109.5
C3—N1—C2	115.2 (2)	H10A—C10—H10C	109.5
C9—N2—C11	121.8 (3)	H10B—C10—H10C	109.5
C9—N2—C10	122.8 (3)	C16—C11—C12	120.5 (3)
C11—N2—C10	115.3 (3)	C16—C11—N2	119.9 (3)
N1—C1—S1	118.6 (2)	C12—C11—N2	119.6 (3)
N1—C1—S2	121.5 (2)	C13—C12—C11	119.7 (3)
S1—C1—S2	119.82 (18)	C13—C12—H12	120.1
N1—C2—H2A	109.5	C11—C12—H12	120.1
N1—C2—H2B	109.5	C14—C13—C12	120.3 (3)
H2A—C2—H2B	109.5	C14—C13—H13	119.9
N1—C2—H2C	109.5	C12—C13—H13	119.9
H2A—C2—H2C	109.5	C13—C14—C15	119.8 (4)
H2B—C2—H2C	109.5	C13—C14—H14	120.1
C8—C3—C4	121.2 (3)	C15—C14—H14	120.1
C8—C3—N1	119.2 (3)	C14—C15—C16	120.5 (3)
C4—C3—N1	119.6 (3)	C14—C15—H15	119.8
C3—C4—C5	119.0 (3)	C16—C15—H15	119.8
C3—C4—H4	120.5	C11—C16—C15	119.3 (3)
C5—C4—H4	120.5	C11—C16—H16	120.4
C6—C5—C4	120.5 (3)	C15—C16—H16	120.4
			
C3—N1—C1—S1	3.9 (4)	C6—C7—C8—C3	−0.1 (5)
C2—N1—C1—S1	−178.4 (2)	C11—N2—C9—S4	−178.2 (2)
C3—N1—C1—S2	−178.5 (2)	C10—N2—C9—S4	−3.1 (4)
C2—N1—C1—S2	−0.7 (4)	C11—N2—C9—S3	2.5 (4)
Cd—S1—C1—N1	−170.8 (2)	C10—N2—C9—S3	177.6 (2)
Cd—S1—C1—S2	11.52 (17)	Cd—S4—C9—N2	174.9 (3)
Cd^i^—S2—C1—N1	86.5 (2)	Cd—S4—C9—S3	−5.81 (16)
Cd—S2—C1—N1	172.4 (2)	Cd—S3—C9—N2	−174.7 (3)
Cd^i^—S2—C1—S1	−95.91 (17)	Cd—S3—C9—S4	5.96 (17)
Cd—S2—C1—S1	−9.98 (15)	C9—N2—C11—C16	−103.8 (4)
C1—N1—C3—C8	83.8 (4)	C10—N2—C11—C16	80.7 (4)
C2—N1—C3—C8	−94.1 (3)	C9—N2—C11—C12	78.8 (4)
C1—N1—C3—C4	−98.0 (3)	C10—N2—C11—C12	−96.7 (4)
C2—N1—C3—C4	84.1 (3)	C16—C11—C12—C13	0.4 (5)
C8—C3—C4—C5	−0.3 (5)	N2—C11—C12—C13	177.8 (3)
N1—C3—C4—C5	−178.4 (3)	C11—C12—C13—C14	−0.5 (5)
C3—C4—C5—C6	−0.1 (5)	C12—C13—C14—C15	−0.2 (5)
C4—C5—C6—C7	0.4 (5)	C13—C14—C15—C16	1.0 (5)
C5—C6—C7—C8	−0.4 (5)	C12—C11—C16—C15	0.4 (5)
C4—C3—C8—C7	0.4 (5)	N2—C11—C16—C15	−177.0 (3)
N1—C3—C8—C7	178.5 (3)	C14—C15—C16—C11	−1.1 (5)

Symmetry code: (i) −*x*, −*y*+1, −*z*+1.

### Hydrogen-bond geometry (Å, º)

*Cg*1 is the ring centroid of the C3–C8 ring.

**Table:**

*D*—H···*A*	*D*—H	H···*A*	*D*···*A*	*D*—H···*A*
C14—H14···*Cg*1^ii^	0.95	2.99	3.883 (4)	156
C5—H5···S1^iii^	0.95	2.75	3.372 (4)	124

Symmetry codes: (ii) *x*+1, −*y*+1/2, *z*−1/2; (iii) −*x*, *y*+1/2, −*z*+1/2.
